# Safety risk assessment of loess tunnel construction under complex environment based on game theory-cloud model

**DOI:** 10.1038/s41598-023-39377-y

**Published:** 2023-07-28

**Authors:** Bing Han, Wei Jia, Weixing Feng, Liu Liu, Zhe Zhang, Yinhu Guo, Mingjie Niu

**Affiliations:** 1 School of Civil Engineering, Shijiazhuang Tiedao University, Shijiazhuang, 050043 Hebei China; 2 The 2th Engineering Company, China Railway 20th Bureau Group Co., Ltd, Xi’an, 710016 Shaanxi China; 3 China Railway First Survey and Design Institute Group Co., Ltd, Xi’an, 710043 Shaanxi China; 4 Xi’an Chengdu Railway Passenger Dedicated Line Shaanxi Co., Ltd, Xi’an, 710045 Shaanxi China

**Keywords:** Engineering, Civil engineering

## Abstract

Due to the impact of the surrounding environment, the safety impact factors encountered during the construction of loess tunnels are complex and numerous, which causes frequent accidents, and there is a lack of safety risk assessment methods applicable to the construction of loess tunnels under complex environment. Based on the Luochuan tunnel project of the Xi’an–Yan’an High-Speed Railway, this paper analyzes the impact factors of loess tunnel construction risks, and 15 main impact factors involving subjective and objective factors are selected to establish the safety risk assessment system of loess tunnel construction under complex environment. To determine the weight of the impact factors, this paper introduces the combination weighting method based on game theory for the first time. Then, the risk assessment model of loess tunnel construction safety is established by using the conventional cloud model theory. Finally, the model is applied to the supporting project for verification. The results show that support and lining have the largest impact on tunnel construction safety, followed by construction management, surrounding rock grade, harmful engineering ground, monitoring measurement, forepoling, and construction method. The assessment result is consistent with the actual construction risk degree, which proves that the assessment result of the model is accurate and reliable, and the model has guiding significance for the safety risk assessment of loess tunnel construction under complex environment.

## Introduction

Loess is widely distributed in the central and western regions of China, and its area reaches 631,000 km^2^, accounting for about 6.6% of the country’s total area^1^. As the Quaternary sediment, loess has the characteristics of vertical joint development, loose structure, and water-softening^2^. The special engineering characteristics also bring a great challenge to the engineering construction in the western region, especially for the tunnel projects crossing the loess stratum, which often encounter disasters such as landslides, projecting mud soil and gushing water, and large deformations during construction, and those disasters causing great safety risks and economic losses^3^. In the past, many scholars have studied the surrounding rock characteristics and the structural stability of loess tunnels^4–6^, and the purpose is to minimize construction risk in production practice. Therefore, the key problem that has to be solved urgently is to thoroughly analyze the construction risks of loess tunnels and propose effective assessment methods.

Numerous academics have studied tunnel risk assessment extensively in recent years. In the book “Guidelines for Tunneling Risk Management” written by Eskesen et al.^7^ risk assessment models and evaluation indexes are discussed, and for the first time, a complete set of reference standards for risk assessment and management in tunneling is proposed. On the basis of a survey research and a systematic summary of more than 50 tunnels, McFeat-Smith and Harman^8^ developed an IMS risk evaluation system that includes many different risk types. Hyun et al.^9^ investigated and analyzed the potential safety risk factors of TBM in the tunnel construction process through Case study and expert consultation, built a fault tree set from three aspects of geology, design, and construction, and effectively predicted the risk occurrence probability and its impact in the TBM tunnel design and construction stages by using FTA and AHP. By analyzing the mechanism of 76 large and medium-sized tunnel collapse accidents, Gao et al.^10^ proposed seven main risk factors as evaluation indexes and realized tunnel collapse risk assessment based on the entropy-weighted-grey system theory. Liu et al.^11^ discussed the relationship between tunnel deformation and human activities, and applies the fuzzy analytic hierarchy process to assess the risk of collapse in loess tunnels under residential areas. Balta et al.^12^ developed a risk identification software for tunnel engineering based on Bayesian theory and successfully applied it to engineering practice. Based on engineering cases and research, Lin et al.^13^ summarized and analyzed four influencing factors closely related to TBM jamming. Using ISM theory, a dynamic BN model was established to obtain the geological conditions in front of the tunnel face and achieve dynamic prediction of geological disasters and TBM jamming during tunnel construction.

In terms of risk assessment methods for underground engineering, including the risk matrix method^14^, risk index method^15^, IMS method^8^, event tree method^16^, fuzzy evaluation method^17^, Bayesian network^18,19^, and neural network^20^, etc. Each method, however, has some limitations and is unable to concurrently account for the great uncertainty and fuzziness of randomness in the tunnel construction process^21^, which cannot guide tunnel construction well in practice. Therefore, many experts and scholars have proposed comprehensive analysis methods for uncertainty based on these theories and methods. Chamzini et al.^22^ considered the problem of fuzziness and randomness, determined the index weights by expert consultation, and established a decision model combined with fuzzy TOPSIS method, providing a new comprehensive evaluation method for TBM scheme selection under the new situation. By identifying the risk factors of the loess tunnel collapse, Zhang et al. established a multi-index evaluation model for loess tunnel collapse risk evaluation by using the rough set theory and extension method^23^. Cai et al.^24^ developed an improved hybrid inference method that simultaneously considers the stochasticity and fuzziness of the risk system to achieve an effective fusion of information from multiple sources. Sharafat et al.^25^ first proposed a risk analysis and management method for TBM tunnelling projects difficult ground conditions based on the generic bow-tie method. By integrating cause and consequence models with fault tree and event tree, they effectively identify and evaluate the risks brought by TBM construction in difficult ground conditions, and provide corresponding remedial measures.

However, most of the previous studies focused on single risk source and static assessment, but the risk events and their impact factors of the same tunnel in different times and spaces have different sensitivity to the safety risk state. More importantly, the construction process of loess tunnels is significantly affected by the surface environment, tunnel depth and rainfall conditions, and the existing research results do not consider the uncertainty of the evaluation data, which has greater limitations for loess tunnels. Therefore, it is urgent to propose a safety risk evaluation method applicable to the construction of loess tunnels under complex environment.

The cloud model is an uncertainty transformation model proposed by Li that can handle qualitative concepts and quantitative descriptions^26^, which can objectively deal with qualitative and fuzzy problems in the evaluation process and has significant advantages for improving the confidence level of evaluation results. It has been widely applied in various fields such as safety evaluation^27^, decision analysis^28^, risk assessment^29,30^, and has achieved considerable results. However, in the process of tunnel construction risk assessment, the weighting is too dependent on subjective consciousness, resulting in low credibility of the assessment results. As a operations research theory, Game theory can better coordinate the conflicts between different weighting methods, minimize the impact of subjective factors in the weighting of indicators, so as to scientifically and reasonably allocate the weights of various factors^31^.

Based on this, this study aims to propose a new comprehensive risk evaluation model based on the safety of loess tunnel construction under complex environment, which can effectively present the qualitative indicators of complex environment in a quantitative way and help construction units to acquire the risk status of tunnel construction, and optimize the construction plan in time. First, determine the risk evaluation index system and evaluation criteria. Secondly, a new weight fusion model is proposed using game theory to improve the subjective–objective assignment method to determine the weights of each risk indicator. Finally, the standardized cloud model is introduced into the risk assessment of loess tunnel construction to obtain the determinacy of each index under different risk levels, determine the tunnel construction safety risk level by using the maximum subordination degree criterion, and verify the feasibility and effectiveness of the model by comparing the monitoring data of the actual project with the evaluation results. The proposed method can be reliably applied to safety monitoring and early warning of construction risks in loess tunnels, and is equally applicable to other tunnels under complex environment.

## Risk factors identification of loess tunnel construction under complex environment

### Project overview

**Figure 1:**
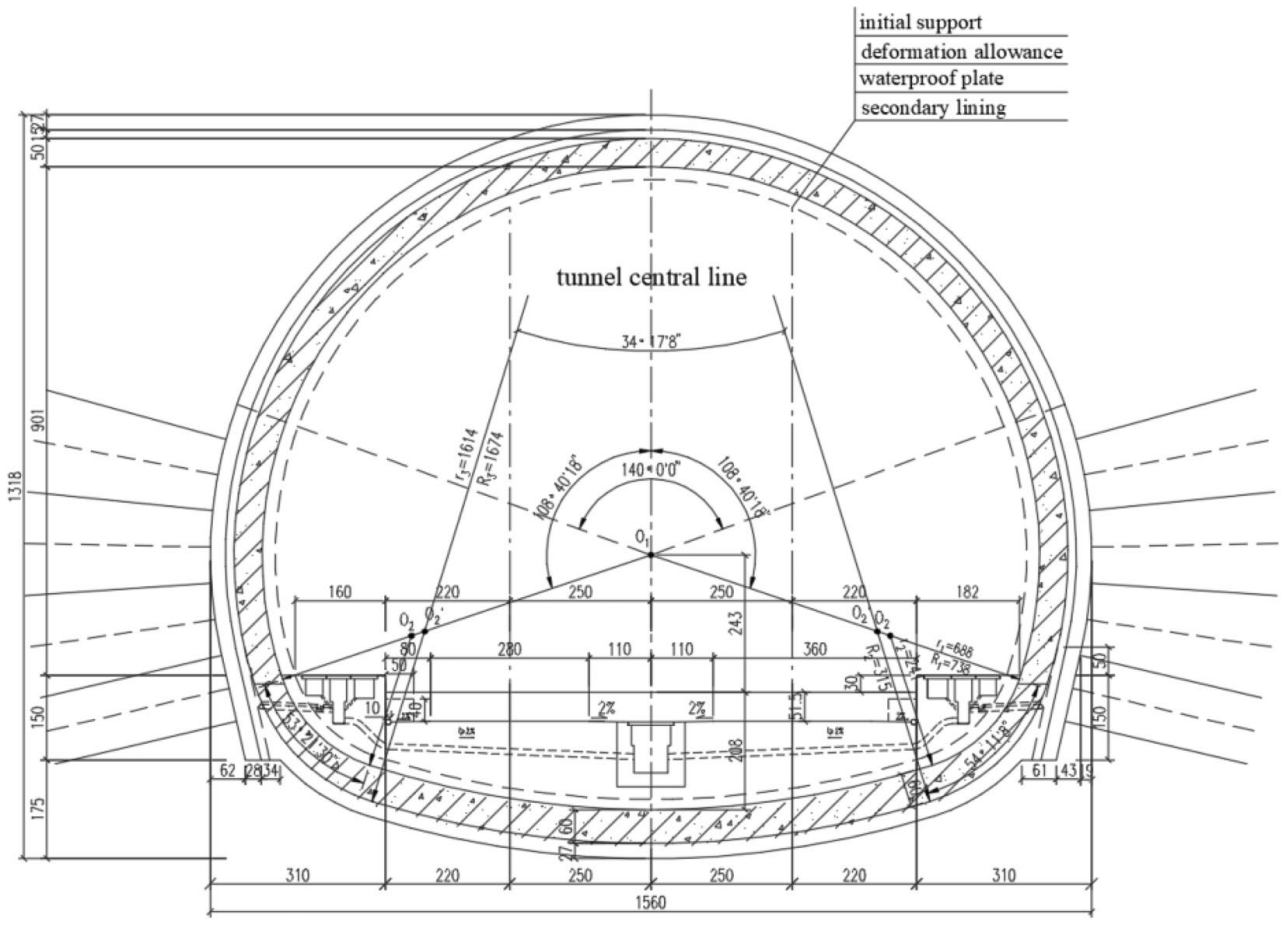
The cross-section of the Luochuan tunnel.

**Figure 2:**
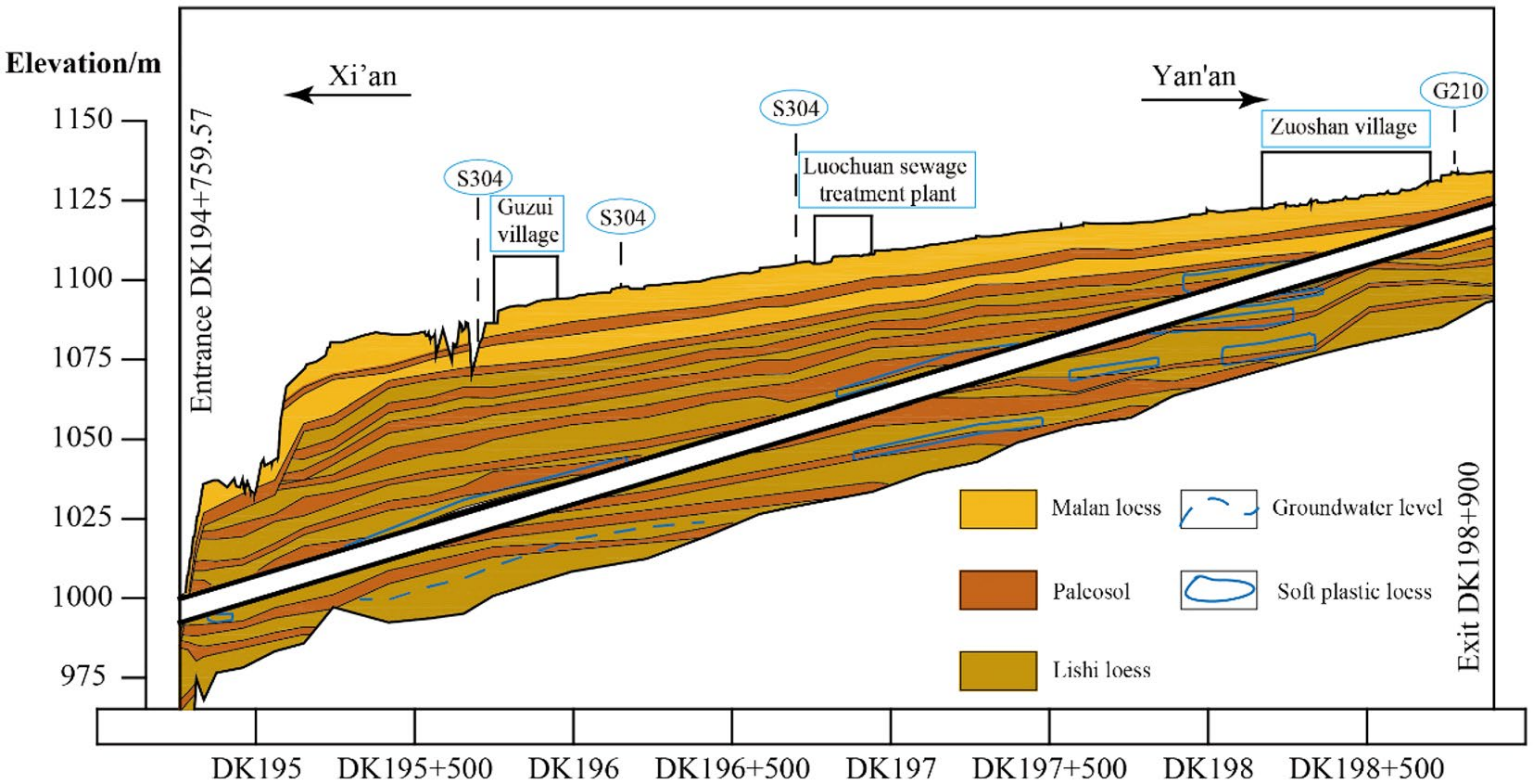
Geological longitudinal section of the Luochuan tunnel.

Located in Luochuan County, Shaanxi Province, the Luochuan Tunnel is a key project of the new Xi’an–Yan’an High-Speed Railway (XYZQ-8 section). The tunnel adopts the design structure of a single-hole double-line railway tunnel, with a total length of 4140.43 m, the theoretical excavation area is 167.3 m^2^ (Fig. 1), and a maximum buried depth of 64 m. The whole tunnel employs the mining method of construction and an inclined shaft is added. The inclined shaft intersects the main line at the mileage DK196 + 700 and has a 53° angle with the line. The tunnel site area belongs to the Weibei Loess Plateau gully area, and the strata that the tunnel crossing are mainly loess. The lithology of the surrounding rock is mainly composed of Quaternary upper Pleistocene Malan loess, Quaternary middle Pleistocene Lishi loess and paleosol. The geological longitudinal section is shown in Fig. 2 and the geotechnical parameters of the rock formations is shown in Table 1.

**Table 1:** Geotechnical parameters.

Stratum	Specific weight (kN/m^3^)	Friction angle (°)	Cohesion (kPa)	Poisson’s ratio	Elastic modulus (kN/m^2^)
Q3 loess	18.1	15	25	0.38	18,000
Q2 loess	19.2	23	36	0.31	46,000

### Construction risks in the engineering area

**Figure 3:**
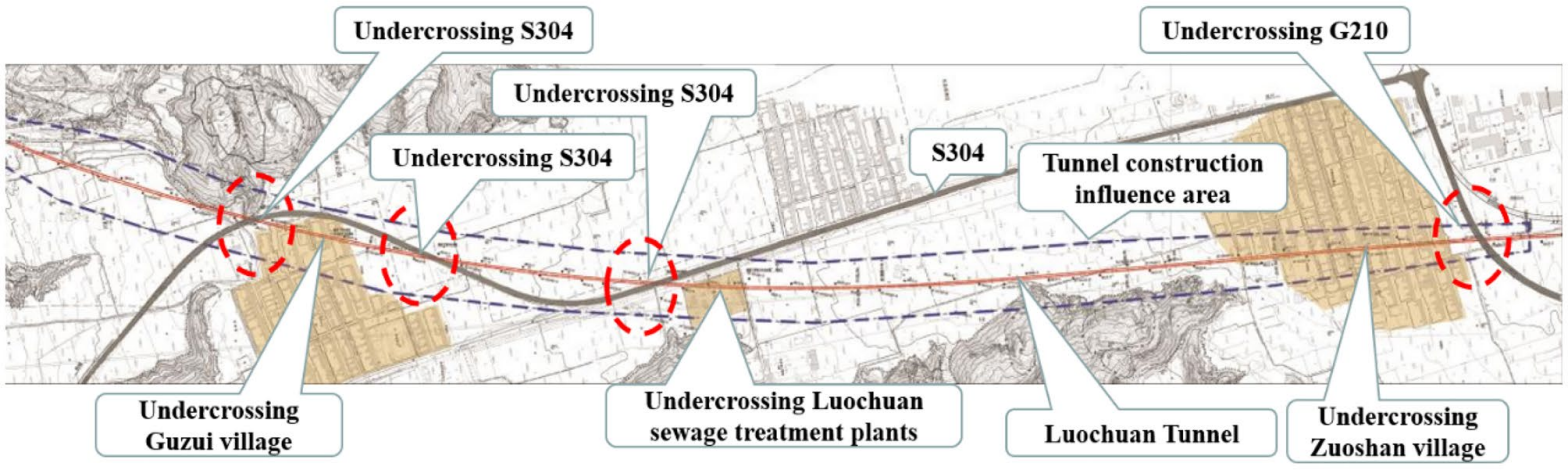
Luochuan tunnel plan.

Luochuan tunnel is the most typical and representative high-risk shallow buried large section loess tunnel among 45 tunnels on the Xi’an–Yan’an railway line, with a complex environment and high construction risk along the line. In terms of geological conditions, the tunnel penetrates through a stratum that contains collapsible loess, soft plastic loess, and expansive paleosol, and the portion of soft plastic loess stratum measures 2073 m in length and is scattered throughout the vault, cavern body, and foundation base. In terms of engineering nature, on one hand, the Luochuan tunnel is characterized by a long shallow buried section, which is 2892 m long, accounting for 70% of the total length, on the other hand, it’s characterized by a complex construction environment, in which the tunnel is under-crossing shallow buried loess gullies and existing structures such as village houses, factories, highways. Additionally, the intersection of the main tunnel and the auxiliary tunnel at the inclined shaft is subject to complex stress. As a result, there are risks of collapse, large deformation, arch falling block, and basement deformation in the tunnel construction, the tunnel plan is shown in Fig. 3.

#### Surface cracking

**Figure 4:**
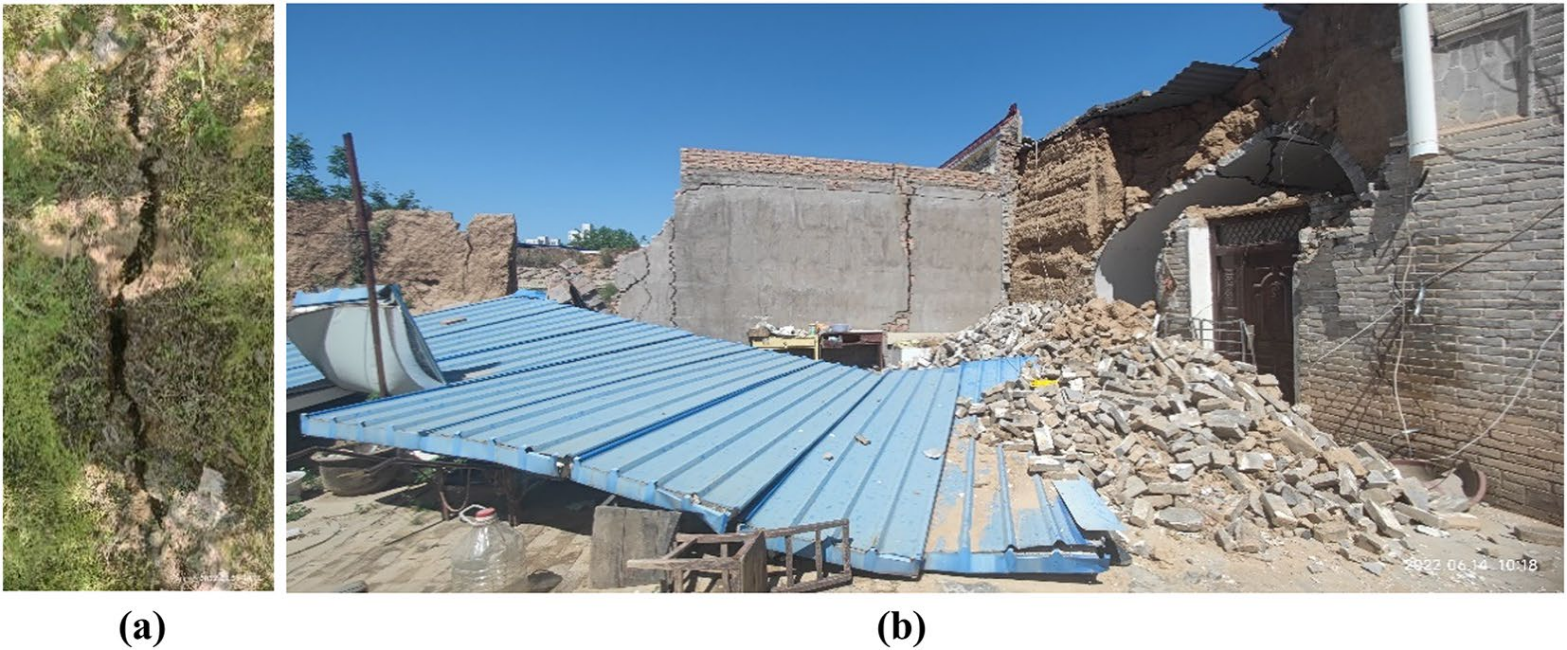
Surface cracks and building damage (**a**) surface cracks, (**b**) building damage.

The buried depth of the underground excavation section DK198 + 170–DK198 + 881 at the exit of Luochuan Tunnel is only 8–18.8 m, and uneven settlement of the ground surface occurred during construction, with the surface range of 50 m on both sides of the tunnel axis is affected by construction. As shown in Fig. 4, ground fissures developed along the tunnel axis on both sides of the tunnel and perpendicular to the tunnel axis are formed above the tunnel, and buildings above the tunnel are also damaged to varying degrees.

#### Large deformation

**Figure 5:**
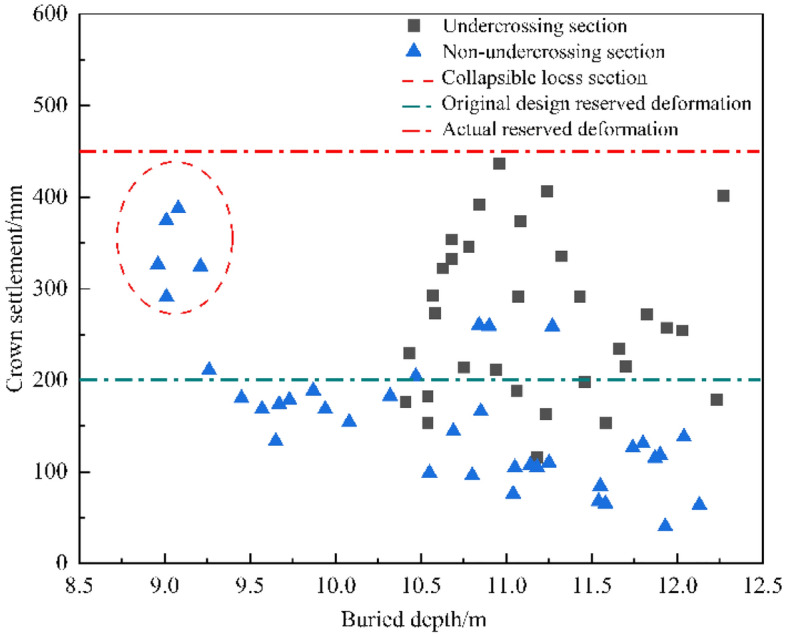
Monitoring results of crown settlement in Luochuan Tunnel.

The exit section of the Luochuan tunnel is distributed with self-weight collapse loess, the section of DK198 + 170–DK198 + 700 undercrossing the Zuoshan village, and large deformation occurred during construction. The monitoring results of arch crown settlement since the construction of the exit section are shown in Fig. 5. It can be seen from Fig. 5 that the deformation of the ordinary shallow buried section is generally in a safe state, and most of the section deformation does not exceed the original design reserved deformation. In the undercrossing section and special geotechnical section, the tunnel deformation exceeds the original design reserved deformation, and the maximum deformation reaches 436.5 mm, far exceeding the original design reserved deformation, which has a great impact on construction safety.

#### Water gushing

**Figure 6:**
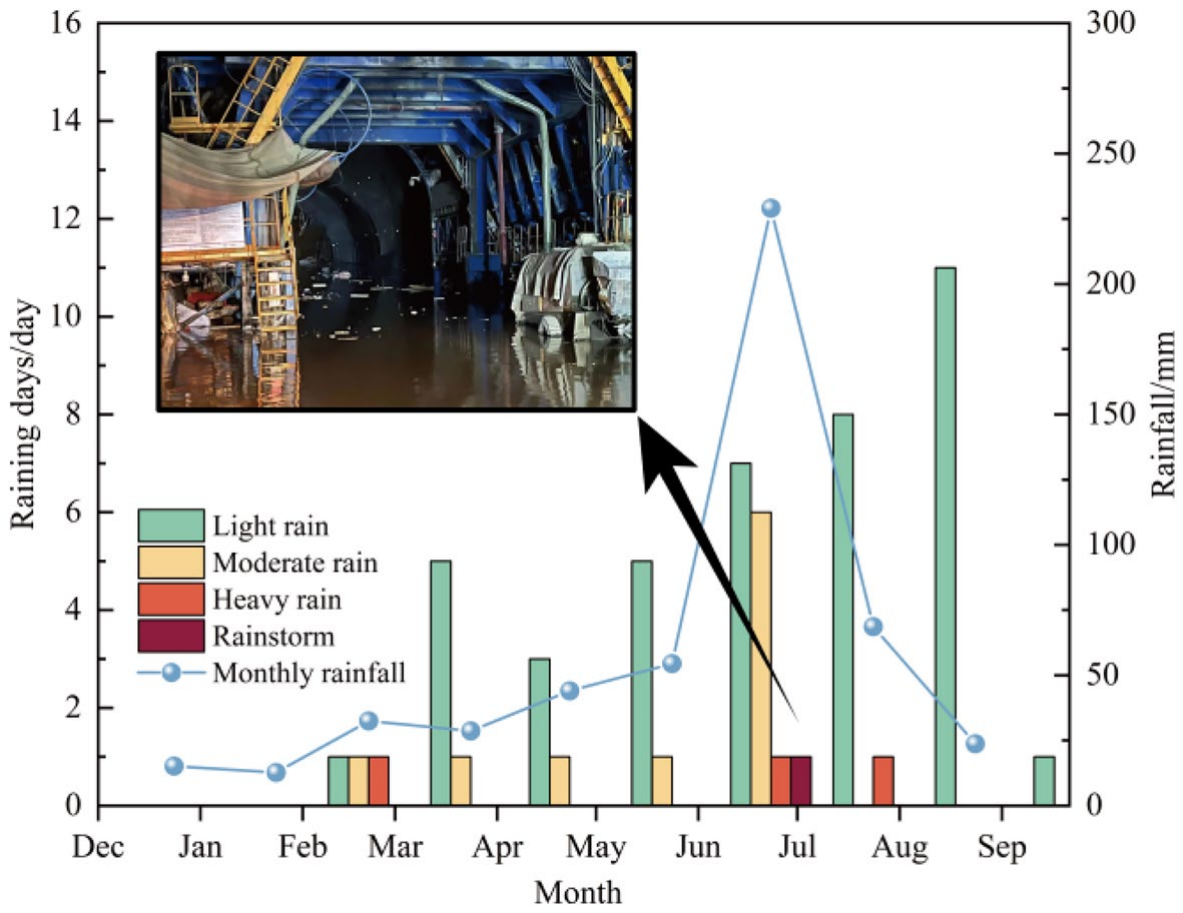
Number of rainfall days and monthly rainfall in Luochuan County (2022).

The tunnel site area belongs to temperate humid continental monsoon climate, with hot summer, concentrated rainfall, and frequent thunderstorms. The annual precipitation can reach 596.7 mm, and precipitation is mainly concentrated from May to September. There are the monthly rainfall days and rainfall since the beginning of construction in 2022 shown in Fig. 6, and it can be seen from Fig. 6 that the maximum rainfall occurs in July. Especially on July 26, the tunnel site area encountered a rainstorm, and rainwater poured into the tunnel along the surface cracks and entrance, causing serious water gushing in the tunnel, which seriously affected the bearing capacity of the foundation base and construction safety.

#### Collapse and block falling

**Figure 7:**
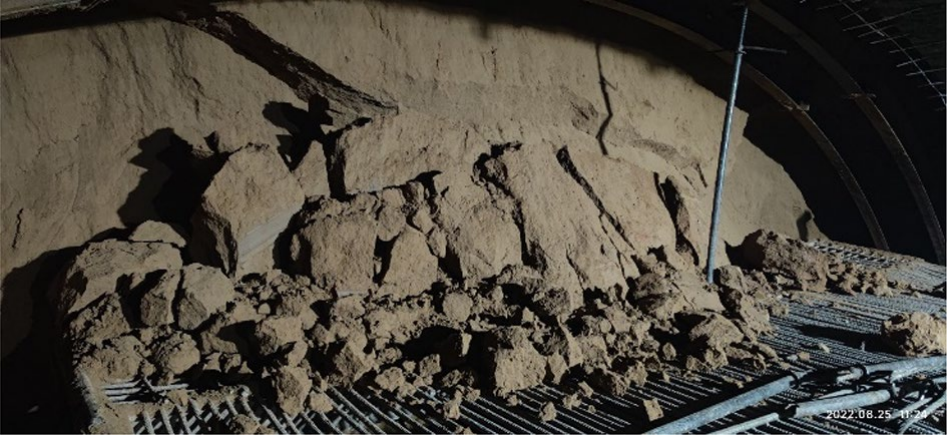
Collapsed block of palm surface.

The clayey loess and paleosol make up the strata that the tunnel crosses, which are primarily composed of silt and have a loose structure. Because of the strong uprightness of loess, the palm surface frequently collapses and falls along its vertical joint surface during excavation, making it very simple to over-excavate the arch. During the actual installation of the steel frame, a safety accident was caused by the collapse of the block, resulting in a worker being injured, as shown in Fig. 7.

The Luochuan tunnel construction risk factors can be categorized into the following four categories as a result of the identification and analysis of the major risk events that occurred during the tunnel’s construction. These risks include landslides, mudslides, water gushing, large deformation, and other disasters. In terms of natural geological conditions, the tunnel crosses harmful geological strata such as self-weighted collapsible loess, the surface is covered with buildings, the rainfall is concentrated in summer, and the surrounding rock is poor, which are the internal factors of the loess tunnel engineering risk accidents. In terms of tunnel design parameters, large tunnel excavation areas and shallow tunnel buried depth are also significant engineering risk factors that result in accidents. In terms of construction technology, forepoling, construction method, support and lining all have a greater impact on construction safety. Therefore, the selection of reasonable construction methods and support parameters is a necessary guarantee to reduce construction risks. Construction management is a direct cause of risky accidents. As an important technical means to ensure construction safety, monitoring and its information feedback are directly related to the occurrence of risky accidents and the safety of construction personnel, while the level of construction organization and management, the technical level of personnel, and the configuration of materials and equipment are indispensable conditions for loess tunnel construction.

### Risk impact factors of loess tunnel construction

Loess tunnel construction risk under complex environment refers to the risk in construction activities under the joint impact of external factors such as surface environment, meteorological conditions, and intrinsic factors such as surrounding rock conditions and construction technology. Based on the risk analysis of Luochuan tunnel construction and the research results of loess tunnel construction risk, the impact factors can be divided into natural geological conditions, tunnel characteristic parameters, construction technology, and safety management.

#### Natural geological condition

Natural geological conditions include the natural environment and the construction environment. The nature of the surrounding rock, meteorological conditions, water yield property, and various harmful geological structures have a significant impact on tunnel construction. Among them, the nature of the surrounding rock is a key factor in the generation of large tunnel deformation. Numerous intricate construction environments, such as undercrossing existing buildings and structures, highways, surface residential activities, and irrigated farmland, are present when loess tunnel construction, which affects the stability of the tunnel's surrounding rock and increases the risk of accidents. Good geological conditions are more conducive to reducing construction safety risks. Logically speaking, the higher the grade of the surrounding rock, the worse the mechanical properties of the rock, the greater the probability of risk in the tunnel. The surrounding rock of the project from IV to VI are distributed.

#### Tunnel characteristics parameters

The area and span of tunnel excavation section are key characteristic parameters of the tunnel construction safety assessment, to a certain extent, they can reflect the difficulty of the excavation and the degree of disturbance to the surrounding rock, which has an important impact on the stability of the tunnel surrounding rock. Most of the loess tunnel excavation sections are not standard circular, therefore, the equivalent tunnel diameter D = 2A/π is used to represent the tunnel section size, where A is the excavation section area. At the same time, the buried depth of tunnel is also a factor that should not be ignored. The weak arching effect of shallow tunnels easily leads to tunnel collapse or large deformation. With the increase of tunnel buried depth, the stable collapse arch is gradually formed, but the concentrated stress at the arch foot and bottom of tunnel also increases.

#### Construction technology

The tunnel has gone through three stages during construction: disturbance damage caused by excavation—dynamic adjustment of support and deformation—final balance and stability. The stability of the surrounding rock is largely dependent on the force and deformation of the support structure, the actual project is divided into different support schemes according to the different grade of the surrounding rock, and the appropriate timing of support is an effective means to control the large deformation of the tunnel. Improper construction is the direct cause of safety accidents in tunnels, including non-conforming excavation techniques, irrational lining support parameters and techniques, late application of support measures, and non-compliant construction operations, which lead to insufficient support strength and is prone to accidents. Therefore, choosing a reasonable construction method and corresponding support system is of great significance for the safe construction of tunnels. The commonly used construction methods include CRD method, CD method, three steps method, and full-section method, and forepoling includes forepoling bolt, large pipe roof, small pipe, etc. It is necessary to dynamically adjust the construction method and support plan according to the continuous changes in geological conditions and mechanical states during tunnel construction to ensure the overall stability of the tunnel during the construction process.

#### Safety management

The factors affecting the safety of tunnel construction are not only the construction scale and environmental conditions of the tunnel, but also the factors of human and management, and safety management is mainly from the perspective of safety technology and organizational management. Monitoring and measurement can grasp the tunnel excavation and support safety status in real-time, forecast and provide early warning of tunnel risk events, and take corresponding measures promptly. In addition, construction management and organization is a multi-factor coupled complicated system with five aspects: human–machine–material–environment–method, which systematically plans, organizes, coordinates, and controls various stages of construction, and directly affects construction safety throughout all phases of the project.

## The proposed model

**Figure 8:**
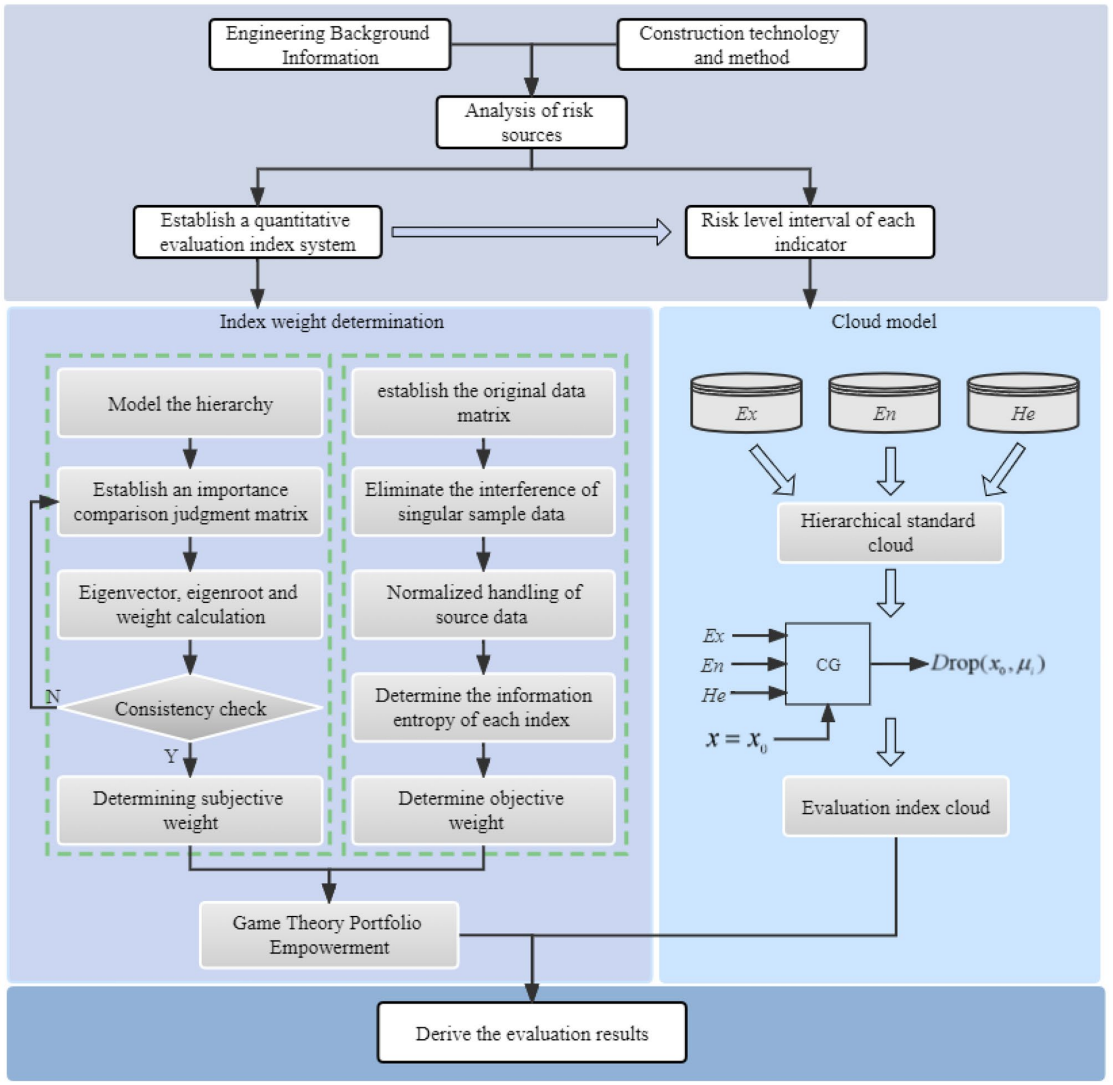
Risk assessment process.

By analyzing the key factors affecting tunnel construction safety, a risk assessment index system for loess tunnel construction is constructed. At the same time, the game theory-cloud model is introduced to determine the optimal weight of each index and construct a comprehensive risk assessment model. Set the system to be evaluated by *U*. The loess tunnel construction safety under complex environment is divided into independent components according to different risk characteristics *U* = (*P*_1_, *P*_2_,…, *P*_m_), and each component contains several subcomponents with different attributes *P*_i_ = (*b*_1_, *b*_2_,…, *b*_n_), where n is the number of evaluation indexes. Figure 8 shows the risk assessment process.

### The establishment of the evaluation index system and the classification of the level

#### Evaluation index system

**Figure 9:**
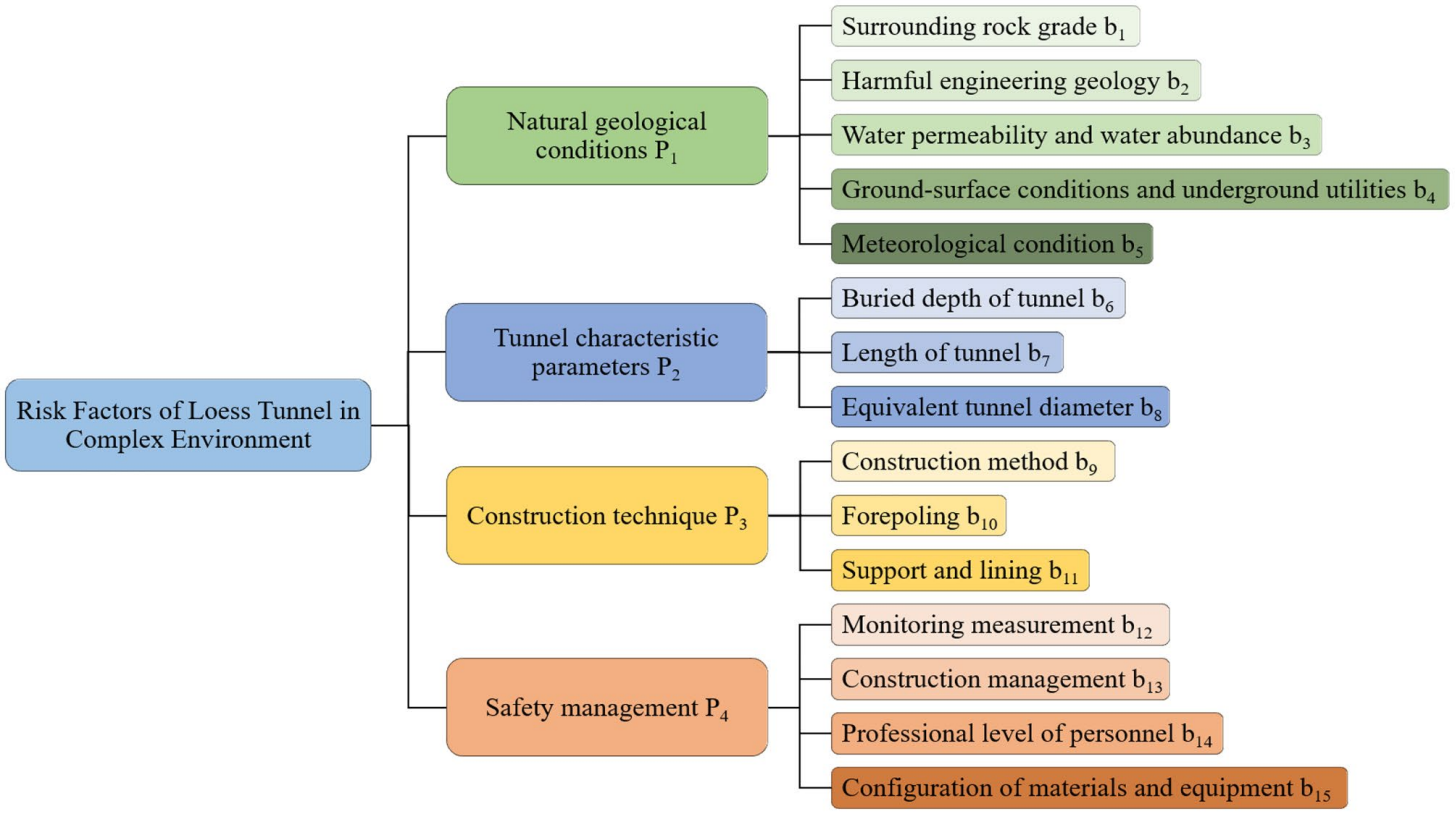
Risk assessment index system chart.

The comprehensive risk assessment index system of loess tunnel construction safety, including 15 indexes such as surrounding rock grade and equivalent hole diameter, is established, as shown in Fig. 9. The rationality of selecting evaluation indicators directly affects the reliability of risk analysis and level discrimination. According to the conclusion drawn from the identification of risk factors, the risk of tunnel construction is the result of the coupling effect of geological factors, design factors, construction factors, and management factors. By analyzing and summarizing the research results of construction risk assessment of representative loess tunnel projects at home and abroad^11,23,32,33^, the system not only draws lessons from the research experiences of experts, takes scientificity, rationality, representativeness, and operability as its establishment principles, but also realizes the combination of dynamic indexes and static indexes.

#### Risk level identification

In view of the current research, there is no consensus on the formulation of safety risk standards for railway tunnel engineering construction. This paper refers to “The Interim Provisions on Risk Assessment and Management of Railway Tunnels”^34^, “The technical Guide for Railway Tunnel Engineering Construction”^35^, and other relevant norms and literature^36–38^. Based on the actual engineering practice, the risk level of loess tunnel construction is defined as five levels from the perspective of the impact scale and hazard level: I (basically no risk), II (low risk), III (medium risk), IV (relatively high risk), and V (high risk), and the risk level interval of each index is shown in Table 2.

**Table 2:** Risk factor classification.

	Risk factors	Risk status description				
		I	II	III	IV	V
Natural geological condition *P*_1_	Surrounding rock grade *b*_1_	–	–	IV (3)	V (2)	VI (1)
	Harmful engineering geology *b*_2_	No harmful geology, no catastrophic (5)	Harmful geology exists, less disaster-causing (4)	Harmful geology exists, moderate disaster-causing (3)	Harmful geological scale is large and more disaster-causing (2)	Harmful geological scale is very large and strongly disaster-causing (1)
	Water Permeability and water abundance *b*_3_	Water-poor area (5)	weakly water-rich area (4)	Medium water-rich area (3)	Strong rich water area (2)	Extremely water-rich area (1)
	Ground-surface conditions and underground utilities *b*_4_	No impact on construction (5)	General impact on construction (4)	Medium impact on construction (3)	Relatively greater impact on construction (2)	Great impact on construction (1)
	Meteorological conditions *b*_5_	No rain or short-time light rain (5)	Light rain or short-time moderate rain (4)	Moderate rain or brief heavy rain (3)	Heavy rain or short-duration rainstorm (2)	Rainstorm or continued heavy rainfall (1)
Tunnel characteristics parameters *P*_2_	Buried depth *b*_6_	≧60	(40, 60]	(20, 40]	(11, 20]	(0, 11]
	Tunnel Length *b*_7_	≦500	(500, 1500]	(1500, 3000]	(3000, 10000]	> 10,000
	Equivalent tunnel diameter *b*_8_	(0, 8.25]	(8.25, 10]	(10, 12]	(12, 14]	> 14
Construction Technology *P*_3_	Construction method *b*_9_	CRD, three-bench temporary inverted arch method or double side drift method (80, 100]	CD or circular excavation pre-set core soil method (75, 80]	Short steps or three steps method (70, 75]	Double-step method (65, 70]	Full-section method (0, 65]
	Forepoling *b*_10_	Reasonable (90, 100]	More reasonable (80, 90]	Moderate (70, 80]	General (60, 70]	Unreasonable (0, 60]
	Support and lining *b*_11_	Reasonable (90, 100]	More reasonable (80, 90]	Moderate (70, 80]	General (60, 70]	Unreasonable (0, 60]
Safety management *P*_4_	Monitoring measurement *b*_12_	Excellent (24, 30]	Good (18, 24]	Moderate (12, 18]	General (6, 12]	Poor (0, 6]
	Construction management *b*_13_	Excellent (32, 40]	Good (24, 32]	Moderate (16, 24]	General (8, 16]	Poor (0, 8]
	Professional level of personnel *b*_14_	Excellent (32, 40]	Good (24, 32]	Moderate (16, 24]	General (8, 16]	Poor (0, 8]
	Configuration of materials and equipment *b*_15_	Excellent (24, 30]	Good (18, 24]	Moderate (12, 18]	General (6, 12]	Poor (0, 6]

Indexes in parentheses are discretized interval assignments of qualitative indexes.

“Harmful geological structure” means whether there are harmful geological zones in the construction area, such as unsymmetrical pressure, gully and sinkhole, earth and stone partition interface, fracture zone, gas stratum, water-rich soft plastic zone, etc. “Ground-surface conditions and underground utilities” refer to whether there are existing buildings and structures, roads, surface residential activities, agricultural irrigation, and other harmful impact factors. “Support and lining” mean whether the lining support parameters and methods are reasonable, whether the support measures are applied promptly, whether the operation meets the requirements, and whether the tunnel over and under excavation is properly handled. “Monitoring and measurement” refer to whether the monitoring and measurement program (monitoring frequency, projects, rules and regulations, personnel allocation) are reasonable, monitoring equipment completeness, and whether the information feedback processing is timely. “Configuration of materials and equipment” refers to the quality inspection and maintenance of machinery and equipment, the efficacy of machinery and equipment, emergency supplies, and equipment security. “The professional level of personnel” including operators, construction managers, and supervisors, the overall quality of the situation, the staffing situation, etc. “Construction organization management” includes construction data integrity, work site management standardization, safety education and training (emergency organization system operation capability), hidden danger investigation, etc.

### Analysis of evaluation index weight

#### AHP

The Analytic Hierarchy Process is a decision-making method proposed by T.L. Saaty for qualitative and quantitative analysis of multi-objective complex problems^39^, which mathematizes the decision-making process of the system based on the concept of multi-factor and multi-level, and constructs a multi-level analysis structure model to determine the relative importance of factors according to the decision maker's experience. In the field of tunnel risk assessment, Hyun et al. used AHP to assess the degree of impact of risks factor on TBM tunnels.

Determination of subjective weight by AHP, whose basic process as follows. Assessing the relative importance of index through pairwise comparison, and the judgment matrix E for each level is constructed by the 1–9 scale method. Calculating the maximum eigenvalue λ_max_ of the judgment matrix satisfying the consistency test, and its eigenvector a represents the weight coefficient. This paper adopts the square root method, as seen is:

1 $$EW = \lambda_{\max } W,$$

2 $$\omega_{i} { = }\sqrt[n]{{\prod\limits_{j = 1}^{n} {a_{ij} } }}{(}j{ = 1,2,}...{\text{,n),}}$$

3 $$\omega_{i} = \frac{{\overline{{\omega_{i} }} }}{{\sum\limits_{i = 1}^{n} {\overline{{\omega_{i} }} } }}{ (}i{ = 1,2,}...{\text{,n)}}{.}$$

Finally, the consistency test is performed on the judgment matrix of each layer, and the test steps are as follows.

Calculate the judgment matrix consistency index:

4 $$CI = \frac{{\lambda_{\max } - n}}{n - 1},$$

5 $$CR = \frac{CI}{{RI}}.$$

If the random consistency ratio *CR* < 0.1, it indicates that the scale selection is reasonable and the result is acceptable; otherwise, return to the assignment to calculate the judgment matrix until the test is passed and the weight value is output.

#### Modified entropy weight method

The entropy value of each risk index is calculated by the entropy weight method. Based on the actual and objective extraction of the implicit information in the index data, The method minimizes the impact of the subjective factors on the determination of the risk index weight^40,41^, so as to obtain more objective evaluation results.

Determination of objective weight by modified entropy weight method is mainly divided into 5 steps:

- *Step 1* Construct the multi-attribute original discriminant matrix M as follows:
  6 $$M = (x_{ij} )_{m \times n} = \left[ {\begin{array}{*{20}c} {x_{11} } & {x_{12} } & \cdots & {x_{1n} } \\ {x_{21} } & {x_{22} } & \cdots & {x_{2n} } \\ \vdots & \vdots & \ddots & \vdots \\ {x_{m1} } & {x_{m2} } & \cdots & {x_{mn} } \\ \end{array} } \right] \, i = 1,2, \cdots ,m;j = 1,2, \cdots ,n,$$
  where *m* is the assessment object; *n* is the assessment index; *x*_*ij*_ is the value of the *j*-th index of the *i*-th object.
- *Step 2* Standardize the judgment matrix to eliminate the interference of odd sample data, and the standardized transformation formula for each risk index is:
  7 $$\left\{ {\begin{array}{*{20}c} {x_{ij}^{*} = \frac{{x_{ij} - x_{\min } }}{{x_{\max } - x_{\min } }}{\text{, Positive indicators}}} \\ {x_{ij}^{*} = \frac{{x_{\max } - x_{ij} }}{{x_{\max } - x_{\min } }}{\text{, Negative indicators}}} \\ \end{array} } \right.,$$
  where *x*_min_ and *x*_max_ are the minimum and maximum values of the *j-*th index respectively, when $x_{\min } = x_{\max }$, take $x_{ij}^{*} = 1$.
- *Step 3* Calculate the contribution of the *i-*th evaluation object of the *j-*th index $p_{ij}$.
  8 $$P_{ij} = \frac{{x_{ij}^{*} }}{{\sum\limits_{i = 1}^{m} {x_{ij}^{*} } }}{ (}i{ = 1,2,} \ldots {,}m{; }j{ = 1,2,} \ldots {,}n{)}{\text{.}}$$
  To make up for the shortcomings of the traditional calculation formula, the calculation formula of entropy weight is modified with reference to the research of Zhang and Ren^42^:
  9 $$p_{ij}{\prime} = \frac{{x_{ij}^{*} + 10^{ - 4} }}{{\sum\limits_{i = 1}^{m} {(x_{ij}^{*} + 10^{ - 4} )} }}.$$
- *Step 4* Determination of information entropy $E_{j}$ for each index.
  10 $$E_{j} = - \frac{1}{\ln m}\sum\limits_{i = 1}^{m} {p_{ij}{\prime} } \ln p_{ij}{\prime} .$$

Among them, $$E_{j} \in \left[ {0,1} \right],\;i = 1,2, \ldots ,m$$.

- *Step 5* Determination of the entropy weight $\omega_{j}$:

11 $$\omega_{j} = \frac{{1 - E_{j} }}{{\sum\limits_{j = 1}^{n} {(1 - E_{j} )} }}.$$

#### Combination weighting model based on game theory

The existing combinatorial weighting models mainly use a single linear weighting or multiplicative synthesis method, while ignoring the consistency and coordination between them. In order to further assign weights scientifically and accurately and obtain optimal weights, game theory is introduced into the weight assignment^43^. The steps to determine the optimal combination weight can be given as in the following:

- *Step 1* The subjective weight $\omega_{1j}$ is determined by the analytic hierarchy process.
- *Step 2* The objective weight $\omega_{2j}$ is determined by the modified entropy weight method.
- *Step 3* The subjective and objective weight algorithms are merged to compensate for the disparity between qualitative and quantitative in the index system. The calculation of the optimal weight is regarded as the game of the two algorithms, and the corresponding linear equations are obtained by minimizing the deviation:

12 $$\left[ {\begin{array}{*{20}c} {\omega_{1} \omega_{1}^{T} } & \cdots & {\omega_{1} \omega_{n}^{T} } \\ \vdots & {} & \vdots \\ {\omega_{n} \omega_{1}^{T} } & \cdots & {\omega_{n} \omega_{n}^{T} } \\ \end{array} } \right]\left[ {\begin{array}{*{20}c} {\alpha_{1} } \\ \vdots \\ {\alpha_{n} } \\ \end{array} } \right] = \left[ {\begin{array}{*{20}c} {\omega_{1} \omega_{1}^{T} } \\ \vdots \\ {\omega_{n} \omega_{n}^{T} } \\ \end{array} } \right].$$

- *Step 4* The resulting optimal linear coefficients are normalized to obtain the final subject-objective combination weights:
  13 $$\alpha_{i}^{\prime} = \frac{{\alpha_{i} }}{{\sum\limits_{i = 1}^{n} {\alpha_{i} } }},$$
  14 $$W = \sum\limits_{i = 1}^{n} {\alpha_{i}^{*} } \omega_{i}^{T} .$$

### Cloud model

#### The fundamental principles

Cloud model is an uncertainty transformation model between qualitative concepts and quantitative descriptions based on fuzzy theory and probability theory proposed by Li et al.^44^ which is widely used in data mining and decision analysis because of its ability to respond to the fuzziness and randomness of objective things. *U* is a quantitative domain represented by exact values, and *C* is a qualitative concept on *U*. If the quantitative value *x* ∈ *U*, and *x* is a single random realization of the qualitative concept *C*, and the certainty degree *µ*(*x*) ∈ [0,1] of *x* for *C* is a random number with a stable tendency, then the distribution of *x* over the domain *U* is said to be a cloud, and each *x* is called a cloud drop. That is:

15 $$\mu :U \to \left[ {0,1} \right],\forall x \in U_{x} \to \mu (x).$$

**Figure 10:**
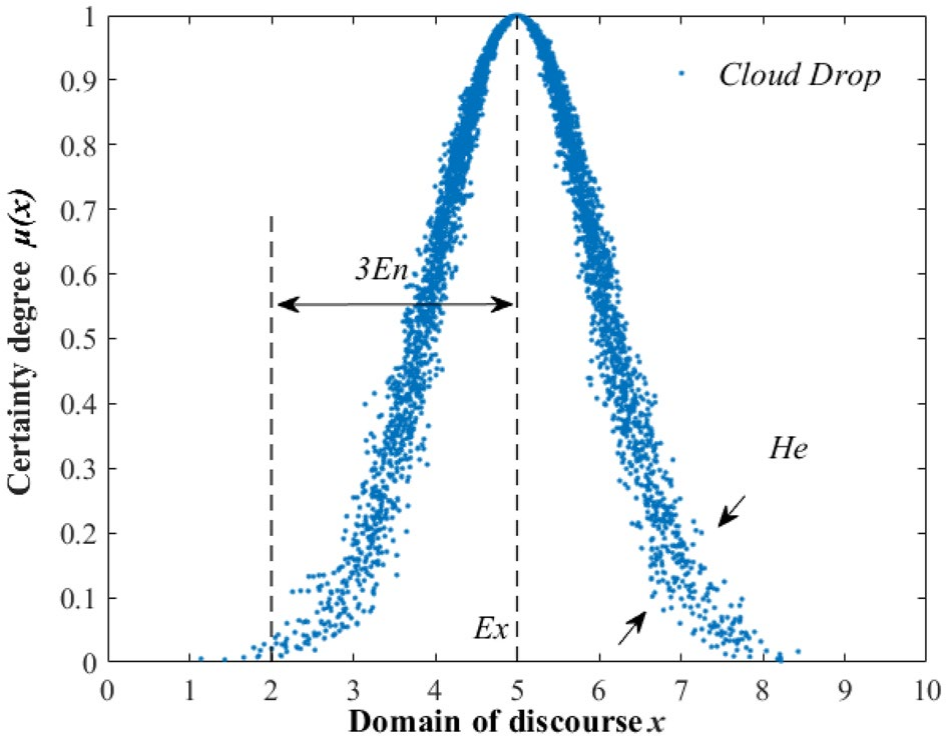
Cloud model characteristic parameters.

The cloud model represents a qualitative concept through the three numerical characteristic parameters: Expectation *E*_*x*_, Entropy *E*_*n*_, and Hyperentropy *H*_*e*_, and the cloud model feature parameters are shown in Fig. 10. These three characteristic parameters can be explained separately as follows.

1. Expectation *Ex* reflects the center of gravity of the statistical cloud drop set, consisting of points in the domain of discourse that best represent the qualitative concept.
2. The entropy *En* represents the uncertainty of the cloud concept, which is a comprehensive measure of the ambiguity and randomness of the qualitative concept, and it reflects the domain degree of character that is both this and that of the qualitative concept.
3. The hyperentropy *He* represents the uncertainty of the cloud entropy, which reflects the degree of cohesion of the “cloud drops” in the cloud model.

The subordinate cloud generator includes a forward cloud generator and an inverse cloud generator. The forward normal cloud generator, which is developed based on the normal distribution and fuzzy mathematics, can realize the process of converting general things from qualitative concepts to quantitative descriptions. By inputting the expectation, entropy, hyperentropy, and the number of cloud drops of the cloud model, the quantitative value of “cloud drops” in the domain of discourse and the degree of certainty of its representation concept can be obtained.

In this paper, we use the forward normal cloud generator, and the conditions that the forward normal cloud generator satisfies is $x\sim N(E_{x} ,E_{n}^{^{\prime}2} )$, which $E_{n}{\prime} \sim N(E_{n} ,H_{e}^{2} )$, and the determinacy of *x* for *C* satisfies:

16 $$\mu = \exp \left( {\frac{{ - (x - E_{x} )^{2} }}{{2\left( {E_{n}{\prime} } \right)^{2} }}} \right).$$

#### Building a risk assessment standard cloud

Building a risk assessment standard cloud is the first task of cloud model evaluation. According to the standard interval of different evaluation levels of each index, its corresponding numerical characteristics of the cloud model (*E*_*x*_, *E*_*n*_, *H*_*e*_) can be calculated according to Eq. (17).

17 $$\left\{ {\begin{array}{*{20}c} {E_{x} = \left( {S_{\max } + S_{\min } } \right)/2} \\ {E_{n} = \left( {S_{\max } - S_{\min } } \right)/6} \\ {H_{e} = kE_{n} } \\ \end{array} } \right.,$$

where *S*_max_ and *S*_min_ represent the critical values of the standard interval of each assessment level respectively. *k* is a constant that controls the domain range of the cloud model, which is taken as 0.1 in this paper.

**Figure 11:**
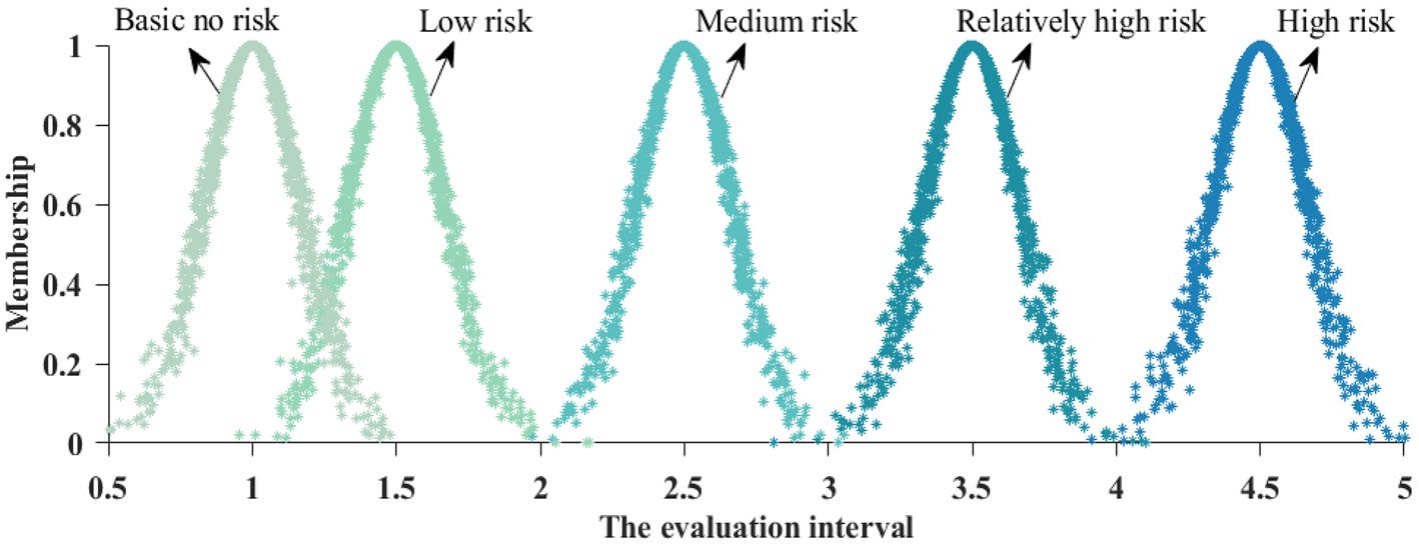
The cloud model of evaluation criteria for natural geological conditions *P*_1_.

If there are unilateral constraints on the value of an index’s quantitative domain *x*_*ij*_, the cloud model characteristic parameters in $(x_{ij}^{1} ,x_{ij}^{2} ]$ can be derived using the calculation method in Table 3. From this, the cloud model characteristic parameters (*E*_*x*_*, E*_*n*_*, H*_*e*_) of each evaluation index in the construction phase of a loess tunnel under complex environment can be determined, and the calculation results are shown in Table 4, and the evaluation standard cloud of a single index is generated by constructing a forward cloud generator using Matlab 2021. The cloud model of evaluation criteria for natural geological conditions *P*_1_ is shown in Fig. 11.

**Table 3:** Determination method of cloud model characteristic parameters.

Characteristic parameters	I	II	III	IV	V
*E*_*x*_	*E*_*x*1_ = (0 + a)/2	*E*_*x*2_ = (a + b)/2	*E*_*x*3_ = (b + c)/2	*E*_*x*4_ = (c + d)/2	*E*_*x*5_ = d
*E*_*n*_	*E*_*n*1_ = (a-0)/6	*E*_*n*2_ = (b-a)/6	*E*_*n*3_ = (c-b)/6	*E*_*n*4_ = (d-c)/6	*E*_*n*5_ = (d-c)/6
*H*_*e*_	0.1*E*_*n*1_	0.1*E*_*n*2_	0.1*E*_*n*3_	0.1*E*_*n*4_	0.1*E*_*n*5_

**Table 4:** Cloud model characteristic parameters of safety risk assessment index.

Risk factors	Cloud model characteristic parameters(*E*_*x*_, *E*_*n*_, *H*_*e*_)				
	I	II	III	IV	V
Surrounding rock grade *b*_1_	(4.5, 0.167, 0.017)	(3.5, 0.167, 0.017)	(2.5, 0.167, 0.017)	(1.5, 0.167, 0.017)	(1, 0.167, 0.017)
Harmful engineering geology *b*_2_	(4.5, 0.167, 0.017)	(3.5, 0.167, 0.017)	(2.5, 0.167, 0.017)	(1.5, 0.167, 0.017)	(1, 0.167, 0.017)
Water permeability and water abundance *b*_3_	(4.5, 0.167, 0.017)	(3.5, 0.167, 0.017)	(2.5, 0.167, 0.017)	(1.5, 0.167, 0.017)	(1, 0.167, 0.017)
Ground-surface conditions and underground utilities *b*_4_	(4.5, 0.167, 0.017)	(3.5, 0.167, 0.017)	(2.5, 0.167, 0.017)	(1.5, 0.167, 0.017)	(1, 0.167, 0.017)
Meteorological conditions *b*_5_	(4.5, 0.167, 0.017)	(3.5, 0.167, 0.017)	(2.5, 0.167, 0.017)	(1.5, 0.167, 0.017)	(1, 0.167, 0.017)
Buried depth* b*_6_	(60, 3.333, 0.33)	(50, 3.333, 0.33)	(30, 3.333, 0.33)	(15.5, 1.5, 0.15)	(5.5, 1.833, 0.183)
Tunnel length *b*_7_	(250, 41.667, 4.167)	(1000, 166.667, 16.667)	(2250, 250, 25)	(6500, 1166.667, 116.667)	(10,000, 1166.667, 16.667)
Equivalent tunnel diameter *b*_8_	(4.125, 1.417, 0.142)	(9.125, 0.292, 0.029)	(11, 0.333, 0.033)	(13, 0.333, 0.033)	(14, 0.333, 0.033)
Construction method *b*_9_	(90, 3.333, 0.333)	(77.5, 0.833, 0.083)	(72.5, 0.833, 0.083)	(67.5, 0.833, 0.083)	(32.5, 10.83, 1.083)
Forepoling *b*_10_	(95, 1.667, 0.167)	(85, 1.667, 0.167)	(75, 1.667, 0.167)	(65, 1.667, 0.167)	(30, 10, 1)
Support and lining *b*_11_	(95, 1.667, 0.167)	(85, 1.667, 0.167)	(75, 1.667, 0.167)	(65, 1.667, 0.167)	(30, 10, 1)
Monitoring measurement *b*_12_	(27, 1, 0.1)	(21, 1, 0.1)	(15, 1, 0.1)	(9, 1, 0.1)	(3, 1, 0.1)
Construction management *b*_13_	(36, 1.333, 0.133)	(28, 1.333, 0.133)	(20, 1.333, 0.133)	(12, 1.333, 0.133)	(4, 1.333, 0.133)
Professional level of personnel *b*_14_	(36, 1.333, 0.133)	(28, 1.333, 0.133)	(20, 1.333, 0.133)	(12, 1.333, 0.133)	(4, 1.333, 0.133)
Configuration of materials and equipment *b*_15_	(27, 1, 0.1)	(21, 1, 0.1)	(15, 1, 0.1)	(9, 1, 0.1)	(3, 1, 0.1)

#### Calculate the determination degree of the safety risk state using the cloud model assessment model

According to the evaluation standard cloud model that has been generated for each index, the x-conditional forward cloud algorithm is used to calculate the certainty degrees of each risk index at different risk levels. The results of comprehensive risk assessment of loess tunnel construction safety under complex environment are obtained by weighted calculation of Eq. (18).

18 $$U_{ki} = \sum\limits_{j = 1}^{m} {\mu_{{\left( {k.ij} \right)}} \omega_{j \, } \, k = 1,2, \ldots ,5,j = 1,2, \ldots ,m} ,$$

where $U_{ki}$ is the integrated degree of certainty of object *i* to be evaluated corresponding to level *k*, $\mu_{{\left( {k,ij} \right)}}$ is the degree of certainty of index *j* in assessment object *i* at level *k*, $\omega_{j}$ is the combined weight of risk assessment indexes.

Normalize the integrated degree of certainty to the object *i* corresponding to level *k*, and the calculation formula is as follows.

19 $$\varphi_{ki} = \frac{{U_{ki} }}{{\sum\limits_{k = 1}^{5} {ki} }},$$

where $\varphi_{ki}$ is the integrated degree of certainty of the *k-*th level after normalization.

20 $${\text{If}}\;U = \max \left\{ {U_{1j} ,U_{2j} , \ldots U_{kj} } \right\}.$$

That is the safety risk level corresponding to the current situation of the object to be measured.

## Engineering study

Taking Luochuan Tunnel of Xi’an–Yan’an High-speed Railway as the research object, the construction environment along the tunnel is complex and variable. There are undercrossing conditions such as roads, factories, residential areas, and gullies, and the tunnel crosses harmful geological layers such as soft plastic loess, collapsible loess, and swelling soil, and the construction process is susceptible to disturbance by external factors such as rainfall, making the construction risk higher and dynamic. According to the recommendations of relevant experts and based on the construction risk analysis of the project area, this paper selects 10 representative sections of the Luochuan tunnel for the construction risk assessment of the loess tunnel under complex environment, the engineering geological conditions and risk factors of each section are shown in Table 5.

**Table 5:** List of section information.

Number	Section mileage	Surrounding rock grade	Buried depth/m	Engineering geological conditions	Risk factors
1	DK194 + 803	V	16	The stratum of the cave is mainly Quaternary aeolian loess, interspersed with multiple layers of paleosol and self-weighted collapsible site at the entrance	Shallow buried section, easily deformed surrounding rock, blocks falling, fall of ground
2	DK194 + 945	V	28.04	The stratum of the cave is mainly Quaternary aeolian loess, interspersed with multiple layers of paleosol, and the soft plastic loess interlayer zone is distributed in the cave body	Shallow buried section, easily deformed surrounding rock, blocks falling, fall of ground
3	DK195 + 095	IV	54.95	The depth of the cave is deeper, and the stratum of the cave is mainly Quaternary aeolian loess, interspersed with multiple layers of paleosol	–
4	DK196 + 680	V	45.7		Undercrossing S304 provincial road
5	DK196 + 760	V	43.73		Inclined shaft intersection section, undercrossing S304 provincial road
6	DK196 + 840	V	43.66	The depth of the cave is deeper, and the stratum of the cave is Quaternary aeolian loess, and the loess at the top of the cave is soft-plastic, interspersed with multiple layers of paleosol	Undercrossing sewage treatment plant
7	DK198 + 558	VI	11.82	The stratum of the cave is mainly Quaternary aeolian loess, interspersed with multiple layers of paleosol	Shallow buried section, undercrossing residential area
8	DK198 + 593	VI	10.96		Shallow buried section in exit, undercrossing residential areas
9	DK198 + 663	VI	10.58		
10	DK198 + 877	VI	9.01	The stratum of the cave is mainly Quaternary aeolian loess, interspersed with multiple layers of paleosol, self-weighted collapsible site at the exit	Harmful geological soil, super shallow buried section

### Determining combined weights

First, concerning the 1–9 scale method introduced in the previous section, a two-by-two comparison of risk categories and indexes of each level of risk factors is performed to establish a two-by-two judgment matrix, and the consistency ratio CI is calculated using an Eq. (4) to discern whether the two-by-two comparison matrix meets the consistency requirements, as shown in Tables 6 and 7.

**Table 6:** The judgment matrix of *P*_1_–*P*_4_.

*P*	*P*_1_	*P*_2_	*P*_3_	*P*_4_
*P*_1_	1	2	2	2
*P*_2_	1/2	1	1/3	1/3
*P*_3_	1/2	3	1	1/2
*P*_4_	1/2	3	2	1
$$\begin{gathered} E = \left\{ {1.682,0.485,0.931,1.316} \right\}^{T} ,\lambda_{\max } = 4.215,CI = 0.072,CR = 0.08 < 0.1, \hfill \\ {\text{meet consistency requirements}} \hfill \\ \end{gathered}$$

**Table 7:** The judgment matrix of *P*_m_–*b*_n_.

*P*_1_	*b*_1_	*b*_2_	*b*_3_	*b*_4_	*b*_5_	*P*_2_	*b*_6_	*b*_7_	*b*_8_	*P*_3_	*b*_9_	*b*_10_	*b*_11_	*P*_4_	*b*_12_	*b*_13_	*b*_14_	*b*_15_
*b*_1_	1	1	4	3	4	*b*_6_	1	2	2	*b*_9_	1	2	1/2	*b*_12_	1	1/2	2	2
*b*_2_	1	1	3	2	3	*b*_7_	1/2	1	1/2	*b*_10_	1/2	1	1/2	*b*_13_	2	1	2	3
*b*_3_	1/4	1/3	1	1	2	*b*_8_	1/2	2	1	*b*_11_	2	2	1	*b*_14_	1/2	1/2	1	2
*b*_4_	1/3	1/2	1	1	3	$$\begin{gathered} E = \left\{ {1.59,0.63,1} \right\}^{T} , \\ \lambda_{\max } = 3.05,CI = 0.03, \\ CR = 0.05 < 0.1, \\ {\text{meet consistency}} \\ {\text{requirements}} \\ \end{gathered}$$				$$\begin{gathered} E = \left\{ {1,0.63,1.59} \right\}^{T} , \\ \lambda_{\max } = 3.05,CI = 0.03, \\ CR = 0.05 < 0.1, \\ {\text{meet consistency}} \\ {\text{requirements}} \\ \end{gathered}$$				*b*_15_	1/2	1/3	1/2	1
*b*_5_	1/4	1/3	1/2	1/3	1									$$\begin{gathered} E = \left\{ {1.19,1.86,0.84,0.54} \right\}^{T} , \\ \lambda_{\max } = 4.07,CI = 0.02, \\ CR = 0.03 < 0.1, \\ {\text{meet consistency}} \\ {\text{requirements}} \\ \end{gathered}$$				
$$\begin{gathered} E = \left\{ {2.17,1.78,0.70,0.87,0.43} \right\}^{T} , \\ \lambda_{\max } = 4.22,CI = 0.07, \\ CR = 0.08 < 0.1, \\ {\text{meet consistency requirements}} \\ \end{gathered}$$																		

According to the calculation steps of AHP, the weight vector of each risk category is calculated as follows: $\omega_{1}^{*} = (P_{1} ,P_{2} ,P_{3} ,P_{4} ) = (0.381,0.11,0.211,0.298)$. Similarly, the weights of the judgment matrix of each risk factor assessment index are $\omega_{11} = (0.365,0.3,0.118,0.146,0.071)$, $\omega_{12} = (0.493,0.196,0.311)$, $\omega_{13} = (0.311,0.196,0.493)$, $\omega_{14} = (0.269,0.42,0.19,0.121)$. The 15 evaluation indexes are weighted layer by layer according to the risk index system structure shown in Fig. 8. The subjective weights of the evaluation indexes can be expressed as:$$\omega_{1} = \left\{ \begin{gathered} 0.1390,\;0.1142,\;0.0448,\;0.0558,\;0.0272,\;0.0543,\;0.0215,\;0.0342,\; \hfill \\ 0.0655,\;0.0413,\;0.1040,\;0.0801,\;0.1253,\;0.0566,\;0.0362 \hfill \\ \end{gathered} \right\}.$$

Secondly, the objective weights are calculated using the modified entropy weight method, and the original discriminant matrix *M*, which consists of the quantified values of the 10 zone evaluation indexes, is normalized by Eq. (7) to obtain the normalized discriminant matrix *N*.

$$N = \left( {x_{ij}^{*} } \right)_{9 \times 15} = \left[ {\begin{array}{*{20}c} {0.5} & {0.5} & 1 & {0.33} & 0 & {0.15} & {0.41} & 1 & 1 & {0.5} & 1 & 1 \\ {0.5} & {0.5} & 0 & {0.67} & 1 & {0.41} & {0.12} & 1 & {0.67} & {0.5} & 1 & 1 \\ 1 & {0.5} & 1 & {0.67} & 1 & 1 & 1 & 1 & 1 & 0 & 0 & 1 \\ {0.5} & 1 & 1 & 1 & 1 & {0.8} & {0.41} & 1 & 1 & 0 & {0.67} & 0 \\ {0.5} & 0 & 0 & 0 & 1 & {0.76} & {0.12} & 0 & {0.33} & 0 & {0.67} & 0 \\ {0.5} & 0 & 0 & 0 & 1 & {0.75} & {0.71} & {0.33} & 0 & 0 & 0 & 0 \\ 0 & 0 & 1 & {0.33} & 1 & {0.06} & 0 & {0.67} & {0.33} & 1 & 0 & 0 \\ 0 & 0 & 0 & 0 & 0 & {0.04} & 0 & {0.33} & 0 & {0.5} & 0 & 0 \\ 0 & 0 & 0 & {0.33} & {0.75} & {0.03} & 0 & {0.33} & 0 & {0.5} & 0 & 0 \\ 0 & 0 & 0 & 0 & 1 & 0 & 0 & {0.33} & 0 & {0.5} & 0 & 0 \\ \end{array} } \right].$$ Referring to steps 3 to 5 of “Modified entropy weight method” section, the objective weights of each index can be obtained. $$\begin{gathered} \omega_{2} = (0.0577,0.0111,0.1041,0.0644,0.0328,0.0501, \hfill \\ 0.0000,0.0000,0.0828,0.0284,0.0641,0.0817,0.1066,0.0755,0.1410) \hfill \\ \end{gathered}$$.

Finally, using Eqs. (12) and (14) to obtain the weights of the optimal index combination based on game theory, the results are shown in Table 8.

**Table 8:** Game theory determination of portfolio weights.

Risk factors	Subjective weights	Objective weights	Portfolio weights
Surrounding rock grade *b*_1_	0.1390	0.0577	0.0937
Harmful engineering geology *b*_2_	0.1142	0.0111	0.1124
Water permeability and water abundance *b*_3_	0.0448	0.1041	0.0778
Ground-surface conditions and underground utilities *b*_4_	0.0558	0.0644	0.0606
Meteorological conditions *b*_5_	0.0272	0.0328	0.0303
Buried depth *b*_6_	0.0543	0.0501	0.0520
Tunnel length *b*_7_	0.0215	0.0000	0.0095
Equivalent tunnel diameter *b*_8_	0.0342	0.0000	0.0152
Construction method *b*_9_	0.0655	0.0828	0.0751
Forepoling *b*_10_	0.0413	0.0284	0.0341
Support and lining *b*_11_	0.1040	0.0641	0.0818
Monitoring measurement *b*_12_	0.0801	0.0817	0.0810
Construction management *b*_13_	0.1253	0.1066	0.1149
Professional level of personnel *b*_14_	0.0566	0.0755	0.0671
Configuration of materials and equipment *b*_15_	0.0362	0.1410	0.0945

### Determine the level of safety risk assessment

**Figure 12:**
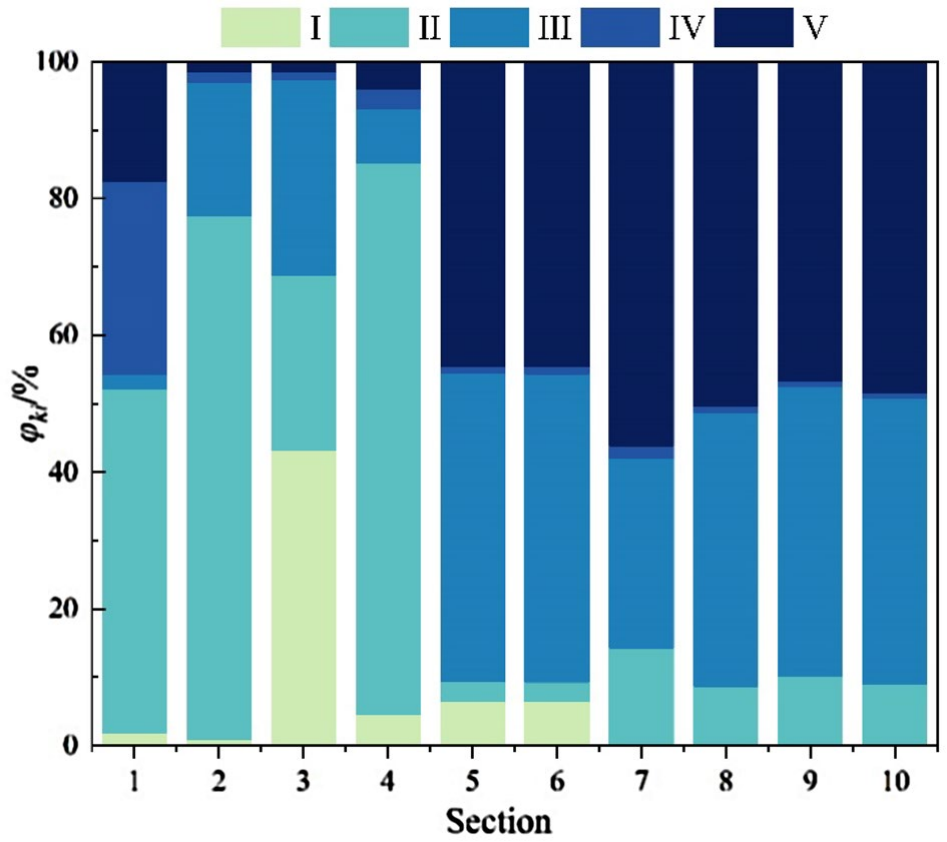
Normalized integrated certainty.

According to the parameters of the cloud model for each risk level identified in Table 4, using Matlab and the formula to substitute the measured and quantified values of the corresponding indexes into the x-condition generator in the cloud model for calculation (N = 3000), and finally derive the cloud model for each index assessment. Combined with the weight values of each index combination in Table 8, the evaluation model can be derived by applying the Eqs. (17) and (18), and the normalized integrated certainty of each section is shown in Fig. 12.

**Figure 13:**
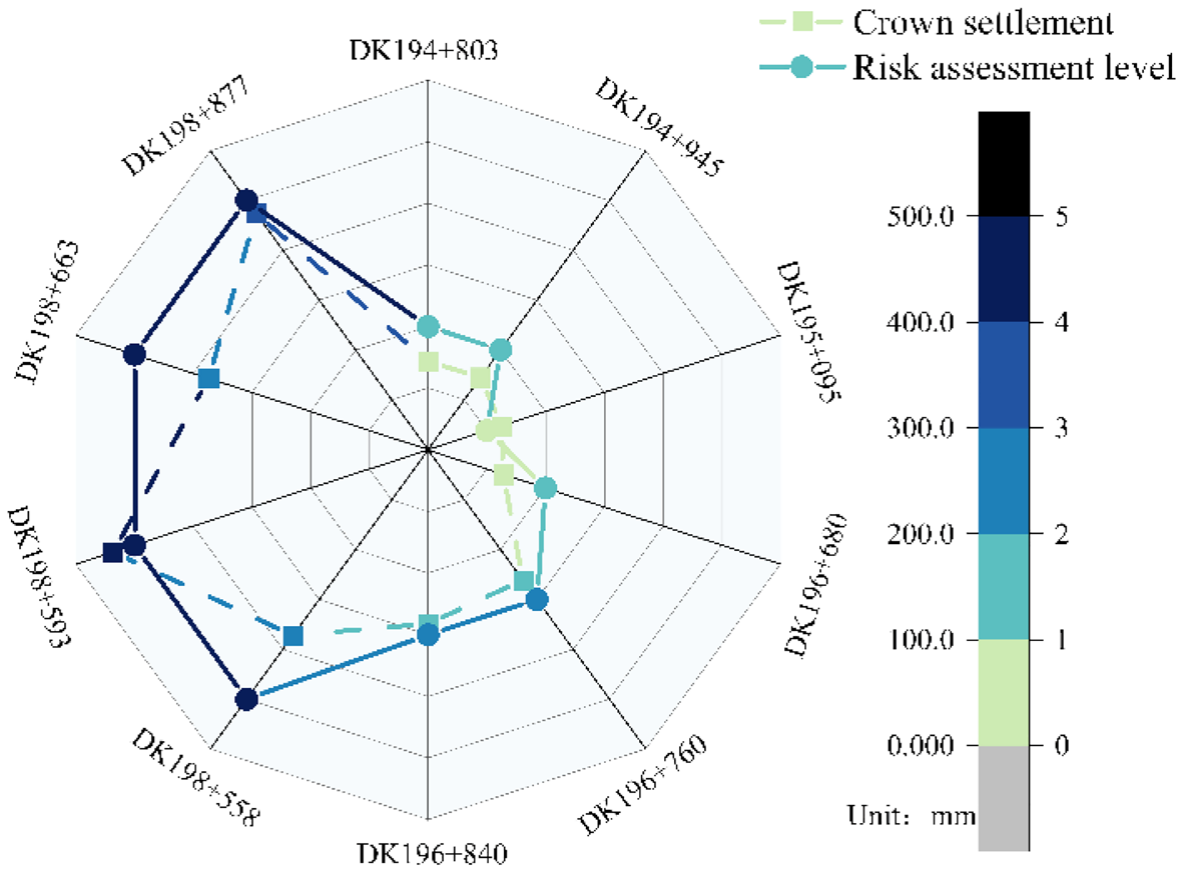
Risk assessment results and actual deformation amount.

According to Eq. (20), the construction safety risk level of each study area can be determined, and the safety risk level assessment results and measured deformation of each section are shown in Fig. 13.

### Result analysis and validation

The deformation amount is a visual representation of the stability of the support structure, and the deformation of the loess tunnel is mainly the overall sinking, so the crown settlement can be used as a criterion of safety during the construction of the loess tunnel, which reflects the level of construction risk to a certain extent. Compare the risk assessment level of each section with the actual deformation amount, as can be seen from Fig. 13, the distribution trend of the risk assessment level of each section is consistent with that of the actual deformation amount. Among them, the DK198 + 877 section is a super shallow buried section covered by collapsible loess, the deformation amount of this section exceeds the original design reserved deformation amount, and the section construction risk is level V. DK198 + 593 section crown settlement reaches 436.5mm, at the surface of the section, the formation of the settlement trough creates conditions for rainfall polling, and the surface cracks become the dominant channel for infiltration. When the tunnel encountered rainstorm weather on July 26th, a large amount of mud and water poured into it, resulting in block falling and deformation intrusion. The construction risk of this section is level V, consistent with the actual situation. DK198 + 663 and DK198 + 558 are located in the section of the undercrossing residential area where the primary support has large deformation, and the risk assessment is level V, which is consistent with the actual situation. DK196 + 840 and DK196 + 760 are located in the cross-section of the inclined shaft and section of the undercrossing factory respectively, both of them have a large buried depth, the surrounding rock condition is grade V, the risk assessment level is III, the actual deformation is within the control range. While DK198 + 680 has a higher support strength and risk assessment level is II, which is in line with the actual construction. The deformation of the rest of the section is small, and the risk assessment level is not higher than II. The risk assessment grade of the above samples is consistent with the actual risk situation on the construction site, which testify the availability of the model.

For this project, the main reasons for the high risk level of tunnel construction are as follows. (1) The engineering characteristics of the loess itself lead to poor stability of the surrounding rock and high construction difficulty. (2) The tunnel has a large cross-section under complex construction environment, which greatly affects the mechanical properties of the surrounding rock when crossing soft-plastic loess layer for a long distance. (3) Delayed design alteration. (4) Weaknesses remain in the tunnel construction safety management system, including untimely monitoring and measurement, and the lack of special emergency plans for risk sources. Therefore, the subsequent construction should take measures to improve the geotechnical properties of the soil around the excavation surface, such as advanced small pipe grouting, soil reinforcement, and additional waterproof strip cloth on the surface. In the construction of high-risk sections, the support strength should be appropriately increased and the monitoring feedback should be strengthened so as to take corresponding measures on time to ensure the safety of tunnel construction.

## Conclusions

Loess tunnel construction under complex environment has many construction impact factors, a large impact on society and other characteristics, and it belongs to high-risk engineering, To reduce the risk of construction accidents, this paper proposes a novel risk assessment method for loess tunnel construction based on game theory-cloud model theory, with clear analysis process and reliable results, which strongly guidance for design and construction, and providing reference for the implementation of similar projects. The main conclusions are as follows.

1. A comprehensive assessment index system of loess tunnel construction safety risk under complex environment is constructed in four aspects by analyzing the various factors affecting the safety of loess tunnel construction: natural geological conditions, tunnel characteristic parameters, construction technology, and safety management, and concerning existing research results at home and abroad, the assessment levels are divided into five levels: basic no risk (level I), low risk (level II), medium risk (level III), relatively high risk (level IV), and high risk (level V).
2. A novel weight fusion model is proposed by the existing hierarchical analysis method and the modified entropy method are integrated on the basis of the pertinent game theory to realize the natural conversion of qualitative indexes and quantitative data, which greatly removes the subjective uncertainty of decision-makers.
3. Applying the EAHP-cloud model to the comprehensive assessment of loess tunnel construction safety risk under complex environment can better deal with the ambiguity and high uncertainty in the assessment process, which provides an effective method for loess tunnel construction safety risk assessment and provide reliable decision support for managers.
4. The proposed method can effectively predict the safety risk status of tunnel construction and take measures in advance to reduce construction safety risks. In addition, the method can also be used in other tunnel projects, but each application must take into account the unique aspects of the project in order to make the necessary adjustments for each individual special risk indicator of the weight distribution and evaluation, so as to maximize compliance with engineering practice and effectively leverage the reliability of the evaluation method.

## Data Availability

The datasets generated during and/or analyzed during the current study are not publicly available but are available from the corresponding author on reasonable request.
